# Inter- and Intra-observer Agreement of the Peripheral Arterial Calcium Scoring System in Patients Undergoing (Infra)Popliteal Endovascular Interventions

**DOI:** 10.1007/s00270-024-03839-1

**Published:** 2024-08-26

**Authors:** Michael J. Nugteren, Çağdaş Ünlü, Morsal Samim, Hester J. Scheffer, Gert J. de Borst, Constantijn E. V. B. Hazenberg

**Affiliations:** 1 Department of Vascular Surgery, University Medical Center Utrecht, Heidelberglaan 100, 3584CX Utrecht, The Netherlands; 2 Department of Vascular Surgery, Noordwest Ziekenhuisgroep, Alkmaar, The Netherlands; 3 Department of Radiology, University Medical Center Utrecht, Utrecht, The Netherlands; 4 Department of Radiology, Noordwest Ziekenhuisgroep, Alkmaar, The Netherlands

**Keywords:** Peripheral artery disease, Infrapopliteal, Popliteal, Vascular calcification, Observer agreement

## Abstract

**Purpose:**

Peripheral arterial calcification is an important predictor of outcomes after both conservative and endovascular treatment. Digital subtraction angiography (DSA)-based calcification scores are limited by low sensitivity and inter-observer agreement. The Peripheral Arterial Calcium Scoring System (PACSS) assesses the severity of target lesion calcification. The newly introduced modified PACSS (mPACSS) also evaluates target vessel calcification. This study aimed to assess the inter- and intra-observer reliability of PACSS and mPACSS on computed tomography angiography (CTA) in (infra)popliteal endovascular interventions.

**Methods:**

A random sample of 50 limbs from the prospective multicenter Dutch Chronic Lower Limb-Threatening Ischemia Registry (THRILLER) were included. Three experienced independent raters scored PACSS on CTA. Three months later, one blinded rater assessed the same 50 CTA scans, keeping track of assessment time. The reliability of the original 5-step PACSS, a simplified binary PACSS (0–2 vs 3–4) and the 7-step mPACSS were tested using Cohen’s and Fleiss’ kappa statistics.

**Results:**

In total, 50 limbs (mean age 70.1 ± 11.0, 29 men) with 41 popliteal and 40 infrapopliteal lesions were scored. Inter-observer agreement of PACSS and binary PACSS were moderate (*κ* = 0.60) and substantial (*κ* = 0.72), respectively, while intra-observer agreement was almost perfect in both scores (*κ* = 0.86). Inter- and intra-observer agreement of mPACSS were moderate (*κ* = 0.48) and substantial (*κ* = 0.77), respectively. Mean assessment time for an experienced rater was 3.43 ± 0.93 min per CTA scan.

**Conclusion:**

Both the semi-quantitative PACSS and mPACSS scores for (infra)popliteal arteries can be performed reliably on pre-operative CTA.

**Graphic Abstract:**

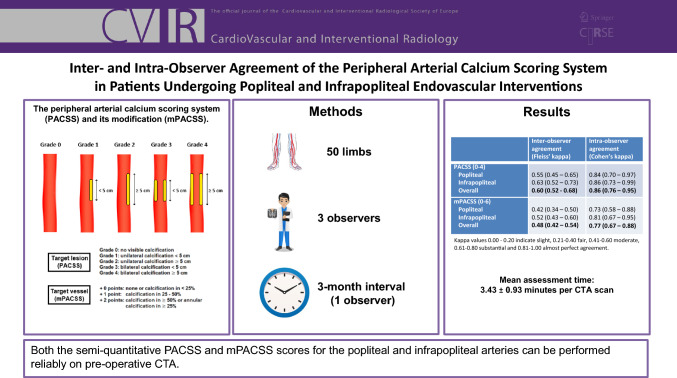

**Supplementary Information:**

The online version contains supplementary material available at 10.1007/s00270-024-03839-1.

## Introduction

The global prevalence of peripheral arterial disease (PAD) is increasing, with a particular rise in the proportion of patients with chronic limb-threatening ischemia (CLTI) [1]. This has resulted in an increasing demand for below the knee (BTK) revascularizations, which can be challenging due to long lesion length, small vessel diameter and severe calcification [2].

In the field of PAD, severe calcification complicates endovascular interventions because it causes difficulties to cross the lesion, leads to early elastic recoil and incomplete expansion of stents, and forms a mechanical barrier for drug penetration when using drug-eluting devices [3]. In previous studies, severe calcification was associated with an increased risk of technical failure, loss of patency, clinically driven target lesion revascularization (CD-TLR), major adverse limb events (MALE), amputation and mortality in both conservative patients and after endovascular intervention [4–15].

Multiple quantitative and semiquantitative peripheral calcification scores have been developed and used in clinical studies and trials [4–15]. The most commonly used scores, the semiquantitative peripheral arterial calcium scoring system (PACSS) and peripheral academic research consortium (PARC), are scored on two-dimensional digital subtraction angiography (DSA) imaging [3, 16]. The accuracy and reliability of these scores are important for the interpretation of trial outcomes that depend on the severity of calcification, such as device trials. However, previous studies concluded that DSA is limited in its ability to identify calcium and the inter-observer agreement of both PACSS and PARC was minimal [16, 17]. These studies also mention that methods using cross-sectional imaging are warranted and that computed tomography angiography (CTA)-based systems may outperform two-dimensional DSA-based systems.

Lastly, it is relevant to include the calcification of the entire target vessel (TV) rather than just target lesion (TL) calcification [6, 15]. Therefore, we developed the modified PACSS (mPACSS), which integrates calcification of the TL and calcification of the entire TV into one scoring method.

The aim of this study was to assess the inter- and intra-observer reliability of the original and a modified PACSS score based on CTA imaging in popliteal and infrapopliteal endovascular interventions.

## Methods

A random sample of 50 limbs was used from two hospitals that participate in the Du**T**c**H** ch**R**on**I**c **L**ower **L**imb-threatening isch**E**mia **R**egistry (THRILLER). THRILLER is an ongoing national multicenter prospective registry that includes all consecutive patients that undergo a popliteal or infrapopliteal endovascular intervention in 7 Dutch hospitals between February 2021 and December 2023. The protocol was approved by the local ethics committees and was previously published [18]. Informed consent was obtained from all patients. Despite the target population being patients with CLTI, patients were included on the basis of the treatment location (popliteal and the first 2/3 of the infrapopliteal arteries) and not on their clinical manifestation. Exclusion criteria were patients with acute limb ischemia, (infra)popliteal interventions as a result of distal embolization and patients unable to give informed consent.

For this retrospective analysis of prospectively collected data, patients were extracted who underwent a CTA scan before the index procedure. Whether a CTA scan had been performed depended on center and physician preference, indication and the presence of recent imaging. Data regarding patient demographics, comorbidities, medical examinations and imaging, lesion characteristics and procedural characteristics were collected from electronic medical records and entered into an online data capture software.

### Peripheral Arterial Calcium Scoring System

**Fig. 1:**
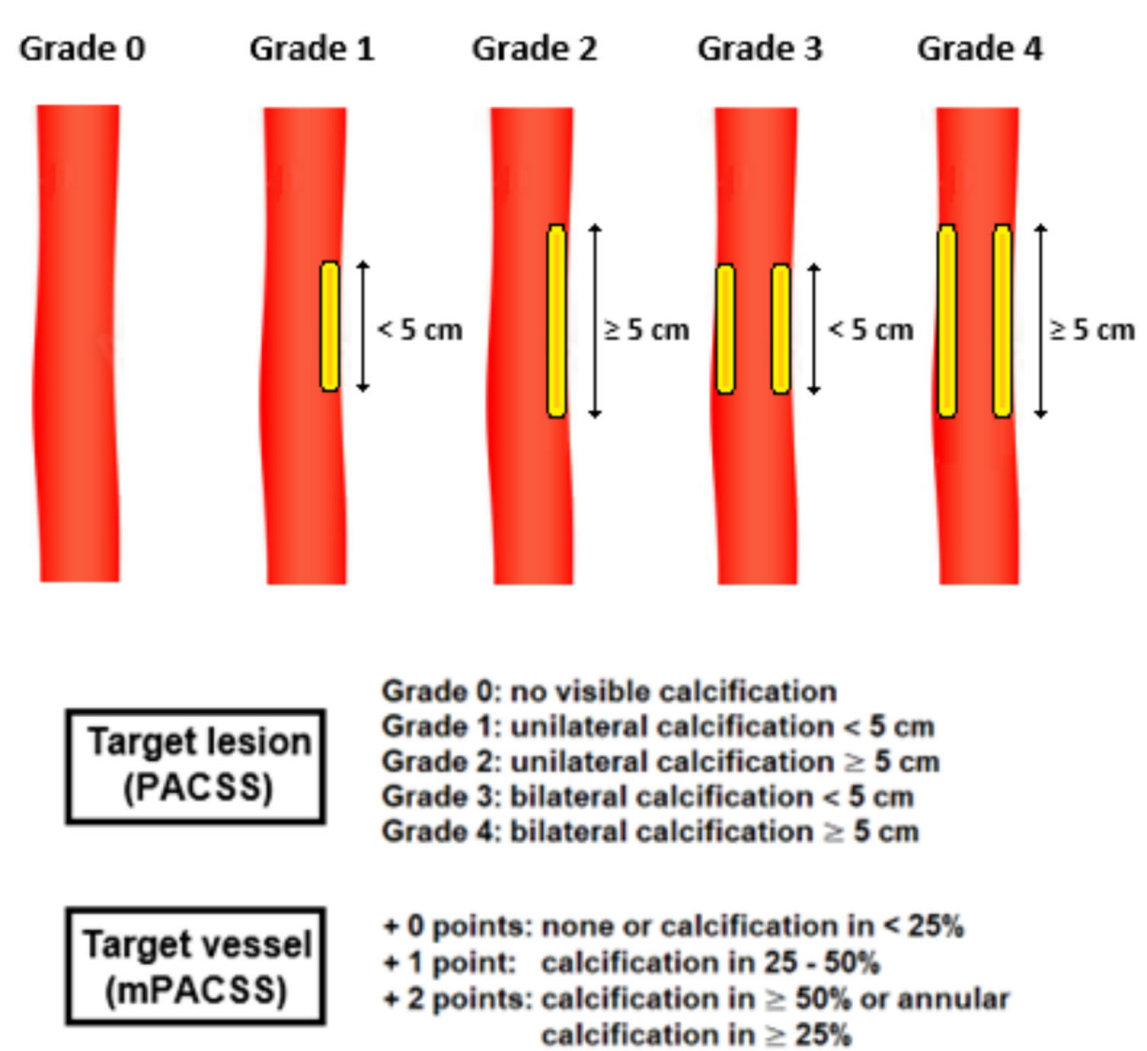
The peripheral arterial calcium scoring system (PACSS) and its modification (mPACSS). The original peripheral arterial calcium scoring system (PACSS) score consists of 5 grades based on the target lesion (TL) calcification. In the mPACSS score, total target vessel (TV) calcification is also scored, resulting in a TV score of 0–2. This score is then added to the existing PACSS score, ultimately giving patients a mPACSS score of 0–6. The TV is the popliteal (POP), tibial anterior (TAA), tibial posterior (TPA) or peroneal artery (PA). In the case the TL is located in both the popliteal and an infrapopliteal artery, both arteries are considered the TV. In the case the TL is located solely in the tibioperoneal trunk (TPT), the TV includes the TPT and the TPA or PA, depending on the target arterial path

**Fig. 2:**
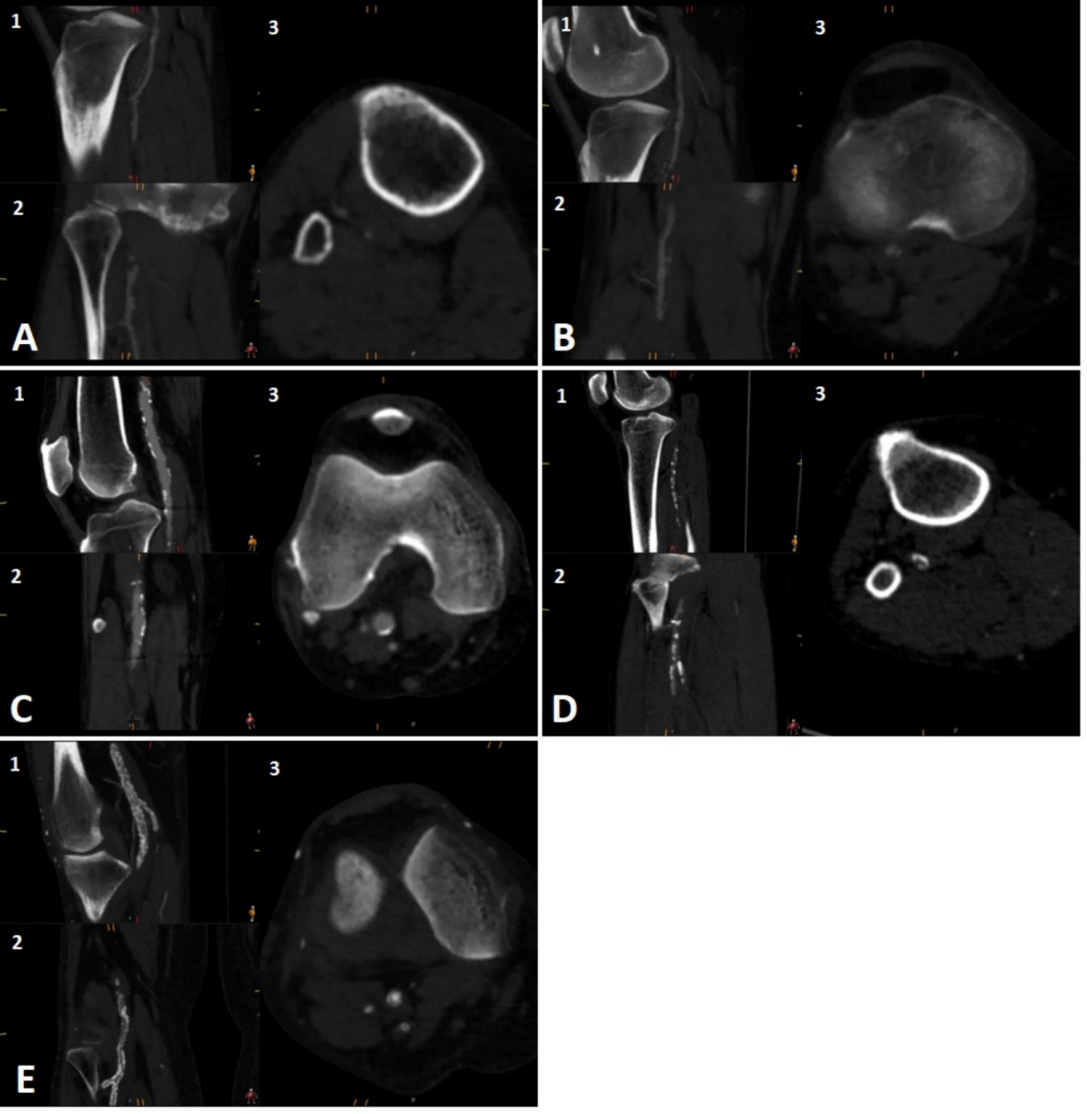
Computed tomography angiography (CTA) examples of the different peripheral arterial calcification scoring system (PACSS) grades. The figure shows sagittal (1), coronal (2) and transversal (3) planes of limbs with PACSS 0 (**A**), PACSS 1 (**B**), PACSS 2 (**C**), PACSS 3 (**D**) and PACSS 4 (**E**)

PACSS was originally designed to be scored on the basis of DSA [3]. However, in this study, all lesions were scored according to the PACSS score based on the preoperative CTA scan between 1 and 1.5 mm slice thickness reconstructions. Bone window settings were used as reference window by all raters and adjusted individually for optimal visualization. Our CTA protocol used a 120 kV with a reference mAs between 100 and 150 depending on patients weight. Solitary lesions in the most distal one-third of the infrapopliteal arteries were not scored, because assessment of circumference in this segments could not be performed reliably. The PACSS score is a semi-quantitative score, meaning that quantitative data is transformed into numerical data to allow for more robust group comparisons. PACSS grades circumference (unilateral vs. bilateral or circumferential) and length (5 cm cut-off) of TL calcification, grading lesions from PACSS 0 to 4 (Figs. 1 and 2). In addition, the original 5-step PACSS score was simplified into a binary score; non-severe calcification was assigned to patients with PACSS grade 0–2 and classified as 0, severe calcification was assigned to patients with PACSS grade 3 and 4 and classified as 1 [9–13, 16].

### Modified PACSS

Because calcification of the entire TV is relevant to predict outcomes after peripheral intervention, an addition was made to the original PACSS score (Fig. 1). In this mPACSS score, total TV calcification is also scored, resulting in a TV score of 0–2. This score is then added to the existing PACSS score, ultimately giving patients a mPACSS score of 0–6. Possible TVs are the popliteal, tibial anterior, tibial posterior (TPA) and peroneal artery (PA). In cases where the TL is located solely in the tibioperoneal trunk (TPT), the TV includes the TPT and the TPA or PA, depending on the target arterial path (TAP).

### Reliability Scoring

Three experienced and independent raters (a vascular researcher and two interventional radiologists) assessed the CTA scans of the included patients (M.N., M.S., H.J.S.). The raters received information regarding the treated limb and lesions, but were blind to all other patient data, procedural information and outcomes. The segments to be scored per lesion on CTA were provided in advance and separately scored according to the PACSS and mPACSS scores. In advance, scoring arrangements were made to improve interobserver agreement (Table 1).

**Table 1:** Arrangements in assessing the (modified) peripheral arterial calcification scoring system (PACSS) score on CTA imaging

Section	Arrangements
General	1. Only popliteal and tibial arteries will be scored (until the ankle joint). Solitary lesions in the most distal one-third of the tibial arteries will not be scored
	2. If available, use transverse images to determine the length and circumference of the calcification
Target lesion	3. The length of the target lesion (TL) equals the length of contiguous stenosis in the peripheral arteries (defined as percent stenosis of diameter> 50%)
	4. Calcification in the target vessel on a different level than the target lesion does not count as lesion calcification
	5. In line with arrangement 4, the length of TL calcification cannot exceed the TL length, i.e. lesions < 5 cm cannot be scored as grade 2 or 4
	6. If the TL consists of multiple calcified plaques, add the lengths of the individual calcified plaques together to determine if the TL calcification is longer than 5 cm
	7. Spotty calcification counts in general as grade 1
Circumference	8. If only a small part of the treated lesion is bilateral, the entire lesion will be scored as bilateral
	9. A bilateral lesion does not necessarily have to be annular
Target vessel	10. In the case the TL is located in both the popliteal and an infrapopliteal artery, both arteries are considered the target vessel (TV)
	11. In the case the TL is located solely in the tibioperoneal trunk (TPT), the TV includes the TPT and the tibial posterior (TPA) or peroneal artery (PA), depending on the target arterial path
	12. If the TV consists of multiple calcified plaques, add the lengths of the individual calcified plaques together to determine the length of TV calcification

To test the intra-observer reliability, one rater (M.N.) assessed the same 50 CTA scans three months after the initial assessment. The rater was blinded to patient data and the initial scores.

### Outcomes

The primary outcomes were the inter- and intra-observer reliability of the original 5-step PACSS score, the binary PACSS score, the TV calcification score and the mPACSS score.

The secondary outcome was the mean time needed to assess one CTA scan for an experienced rater, i.e. the rater that assessed the same 50 CTA scans three months after the initial assessment.

### Statistical analysis

Continuous variables are presented as mean ± standard deviation and categorical variables as absolute number and proportion of the study population. The Cohen’s Kappa (*κ*) statistic was used to test the intra-observer reliability of the PACSS and mPACSS scores. The Fleiss’ Kappa (*κ*) statistic was used to test the inter-observer reliability of both scores. The scale recommended by Landis and Koch was used to interpret the degree of agreement of Kappa results [19]. All statistical analyses were performed using SPSS.

## Results

A total of 44 patients with 50 treated limbs with an available CTA scan prior to an endovascular procedure were included in this study. In total, 41 popliteal and 40 infrapopliteal lesions were treated.

Baseline and lesion characteristics are summarized in Table 2. Diabetes mellitus was present in 54.5% of the patients. A previous target limb revascularization was performed in 58.0% of the limbs. Rutherford categories 3–6 were present in 6.0%, 18.0%, 70.0% and 6.0% of the limbs, respectively. Mean lesion length was 60.9 ± 51.8 mm for popliteal and 150.1 ± 130.6 mm for infrapopliteal lesions, respectively. GLASS 4 was present in 65.9% and 50.0% of the popliteal and infrapopliteal lesions, respectively.

**Table 2:** Baseline and lesion characteristics of the sample population

	Patients (*n* = 44)
*Mean age—years*	70.1 ± 11.0
Male	29 (65.9)
Hypertension	42 (95.5)
Hyperlipidemia	41 (93.2)
Diabetes mellitus	24 (54.5)
*History of smoking*	
Current	13 (29.5)
Previous	29 (65.9)
Coronary artery disease	27 (61.4)
Congestive heart failure	11 (25.0)
Cerebrovascular disease	12 (27.3)
Renal insufficiency (GFR < 30 ml/min)	4 (9.1)
	Limbs (*n* = 50)
Previous target limb revascularization	29 (58.0)
*Rutherford category*	
3	3 (6.0)
4	9 (18.0)
5	35 (70.0)
6	3 (6.0)
*WIfI amputation risk* (*n* = 38)^a^	
1	7 (18.4)
2	11 (28.9)
3	5 (13.2)
4	15 (39.5)

	Popliteal lesions (*n* = 41)	Infrapopliteal lesions (*n* = 40)
*Lesion location*^b^		
ATA		21 (52.5)
PTA		7 (17.5)
PER		4 (10.0)
TPT		13 (32.5)
Mean lesion length—mm	60.9 ± 51.8	150.1 ± 130.6
Total occlusions	12 (29.3)	21 (52.5)
*GLASS stage*		
1	N/A	6 (15.0)
2	6 (14.6)	3 (7.5)
3	8 (19.5)	11 (27.5)
4	27 (65.9)	20 (50.0)

ATA, anterior tibial artery; GFR, glomular filtration rate; GLASS, global limb anatomic staging system; PER, peroneal artery; PTA, posterior tibial artery; TPT, tibioperoneal trunk; WIfI, Wound, Ischemia, foot Infection

^a^In 9 patients the WIfI Ischemia stage could not be calculated due to previous minor amputations or the absence of both a toe pressure and ankle-brachial index

^b^Two lesions were located in the TPT and PTA. Three lesions were located in the TTP and PER

### PACSS Scores

The inter-observer agreement of the original PACSS and binary PACSS scores were moderate (*κ* = 0.60 and substantial (*κ* = 0.72), respectively. The intra-observer agreement of both scores was almost perfect (both: *κ* = 0.86). The inter- and intra-observer agreement of the PACSS score was not significantly different for infrapopliteal lesions compared with popliteal lesions (Table 3).

**Table 3:** Kappa scores divided by a variety of peripheral arterial calcium scoring systems (PACSS) and treatment location

	Inter-observer agreement (Fleiss’ kappa)	Intra-observer agreement (Cohen’s kappa)
*PACSS* (0–4)		
Popliteal	0.55 (0.45–0.65)	0.84 (0.70–0.97)
Infrapopliteal	0.63 (0.52–0.73)	0.86 (0.73–0.99)
Overall	0.60 (0.52–0.68)	0.86 (0.76–0.95)
*Binary PACSS* (0–1)		
Popliteal	0.74 (0.57–0.92)	0.80 (0.61–0.99)
Infrapopliteal	0.70 (0.52–0.88)	0.90 (0.76–1.00)
Overall	0.72 (0.59–0.85)	0.86 (0.74–0.98)
*TV calcification* (0–2)		
Popliteal	0.63 (0.50 – 0.75)	0.85 (0.72 – 0.99)
Infrapopliteal	0.65 (0.50 – 0.80)	0.90 (0.78 – 1.00)
Overall	0.64 (0.54 – 0.73)	0.87 (0.78 – 0.97)
*mPACSS *(0–6)		
Popliteal	0.42 (0.34 – 0.50)	0.73 (0.58 – 0.88)
Infrapopliteal	0.52 (0.43 – 0.60)	0.81 (0.67 – 0.95)
Overall	0.48 (0.42 – 0.54)	0.77 (0.67 – 0.88)

Kappa results should be interpreted as follows: values 0.00—0.20 indicate slight agreement, 0.21–0.40 fair, 0.41–0.60 moderate, 0.61–0.80 substantial and 0.81–1.00 almost perfect agreement

(m)PACSS, (modified) peripheral arterial calcium scoring system; TV, target vessel

### Modified PACSS Scores

The inter- and intra-observer agreement of the TV calcification score were substantial (*κ* = 0.64) and almost perfect (*κ* = 0.87), respectively. The inter- and intra-observer agreement of the mPACSS score were moderate (*κ* = 0.48) and substantial (*κ* = 0.77). The inter-observer agreement of mPACSS was significantly lower for popliteal lesions (*k* = 0.42, 95% CI 0.34–0.50) compared to infrapopliteal lesions (*k* = 0.52, 95% CI 0.43–0.60). No other significant differences were observed between popliteal and infrapopliteal lesions (Table 3).

### Assessment Time

The mean assessment time for an experienced rater was 3.43 ± 0.93 min per CTA scan. The time for scans with one lesion versus scans with multiple lesions was not significantly different (3.29 ± 0.67 vs. 3.56 ± 1.20).

## Discussion

This study showed that scoring PACSS on pre-operative CTA imaging in popliteal and infrapopliteal lesions can be performed reliably if prior scoring arrangements are made. In addition, scoring the calcification of the entire TV appeared to be reliable.

One previous study tested the inter- and intra-observer reliability of semi-quantitative peripheral calcification scores [16]. The referred study scored the PACSS and PARC on DSA and the Fanelli scoring system on both DSA and CTA imaging. All scoring systems were dichotomized and tested on femoropopliteal lesions. The inter- and intra-observer agreement of the binary PACSS were fair (*κ* = 0.32) and moderate to substantial (*κ* = 0.52–0.62) according to the scale recommended by Landis and Koch, respectively. The inter- and intra-observer agreement in the other calcification scores were fair (*κ* = 0.38–0.40) and highly variable (*κ* = 0.36–0.92), respectively.

The PACSS score has been studied primarily in femoropopliteal lesions, in which higher PACSS grades were significantly associated with loss of primary patency, increased target lesion revascularization (TLR), increased MALE and increased mortality [8–12]. Regarding infrapopliteal disease, one retrospective study found that PACSS grade 4 was associated with failed guidewire crossing of below-the-knee chronic total occlusions in univariate analysis [20]. All these studies scored the grade of calcification on two-dimensional DSA instead of CTA imaging. DSA is superior in assessing vessel patency, the absence of which lowers the likelihood of achieving lesion revascularization. However, CTA imaging has the advantages of three-dimensionality and non-invasiveness, and can be performed preoperatively for risk stratification. In addition, previous studies concluded that DSA is limited in its ability to identify calcium as compared with intravascular ultrasound (IVUS). Most importantly, the inter- and intra-observer agreement of PACSS on DSA were unacceptably low [16, 17]. Therefore, these studies raised serious concerns regarding the accuracy and reliability of the currently most commonly used angiographic calcification scores for PAD.

This study demonstrates that the original 5-step PACSS can be scored reliably on CTA imaging. Besides the original PACSS score, we also tested the reliability of a binary PACSS score. Semi-quantitative scores with more than two categories are suitable for determining the prognosis, however, in clinical practice the interventionalist is confronted with a binary question, namely is the calcification present severe enough to choose for an additional calcium modifying treatment such as atherectomy or specialty balloons[16, 21, 22]. The inter-observer agreement of the binary score was slightly higher than the original 5-step PACSS score, while the intra-observer agreement was similarly strong.

In addition, we decided to add the calcification grade of the entire TV, as it might be a more accurate representation of total limb calcification than TL calcification alone, as measured in the original PACSS score. Two studies already demonstrated that TV calcification of infrapopliteal arteries is a significant predictor of technical success, limb salvage amputation-free survival (AFS) and major adverse cardiovascular events (MACE) [6, 15].

The inter-observer agreement of the trichotomic TV calcification and 7-step mPACSS score was substantial (*κ* = 0.64) and moderate (*κ* = 0.48), respectively, while the intra-observer agreement was almost perfect (*κ* = 0.87) and substantial (*κ* = 0.77), respectively. The inter-observer reliability in all scores was calculated with Fleiss’ kappa, which is suitable for calculating the absolute agreement of nominal scores without any intrinsic ordering or ranking [19]. On the other hand, in the case of true ordinal scores, weighted kappa values should be calculated. Regarding PACSS and mPACSS, it is debatable whether these scores should be considered nominal or truly ordinal. In case both scores are considered truly ordinal, the weighted kappa values of inter-observer agreement would be substantially higher (PACSS: *κ* = 0.71*–*0.76, mPACSS: *κ* = 0.72–0.77). Supplementary Table 1 summarizes all weighted kappa values of the PACSS score and its modifications.

Previous studies using CTA imaging to score the grade of infrapopliteal calcification used the quantitative tibial artery calcification (TAC) score and found associations between severe TAC and lower technical success, limb salvage, AFS and survival free of MACE [8, 17]. The inter-observer agreement was high for the quantitative TAC score, which makes quantitative calcium scoring a promising method that deserves further evaluation in future research. On the other hand, quantitative scores are not widely available, time consuming and dependent on a certain level of expertise. This study has shown that semi-quantitative scoring of PACSS on CTA is reliable and fast in popliteal and infrapopliteal disease. Next steps are to test the reliability in femoropopliteal disease and to validate this calcium score on a patient cohort with treatment outcomes, preferably a large prospective multi-center cohort. The use of virtual non-contrast techniques may help improve calcium scoring. Ideally, the scoring system should aid the clinician’s decision in the approach for revascularization, thereby improving revascularization success.

Limitations of this retrospective study are the biased study population, small differences in imaging protocols and limited data regarding intra-observer reliability. In this study, 94% of the limbs were included for CLTI and only popliteal and infrapopliteal lesions were included because the study population was extracted from the THRILLER registry. This may affect the generalizability of the findings of this study to the overall PAD population. Secondly, the protocols for CTA imaging were generally consistent, but minor differences existed. For example, slice thickness varied from 1 to 1.5 mm, which made scoring of calcium bilaterality slightly more difficult in the scans with thicker slices, particularly in the infrapopliteal arteries. Thirdly, only one rater scored PACSS twice, which resulted in limited data regarding intra-observer reliability. However, for this rater, substantial to almost perfect agreement was achieved in all scoring components.

Finally, prior scoring arrangements were essential to improve inter-observer agreement, but may have conflicted with the original assignment of PACSS grades. For example, lesions smaller than 5 cm could not be scored in grades 2 and 4. Nevertheless, the exact interpretation of the different PACSS grades remains unclear and is subject for future scientific research [3].

## Conclusion

This study showed that both the semi-quantitative PACSS and mPACSS scores for the popliteal and infrapopliteal arteries can be scored reliably on CTA. Future studies should focus on the validation of this calcium scoring system in relation to outcomes, possibly leading to improved patient selection and thereby improved revascularization outcomes.

## Supplementary Information

Below is the link to the electronic supplementary material.

Supplementary file1 (DOCX 18 kb)

## Abbreviations

AFS: Amputation-free survival
BTK: Below the knee
CAC: Coronary artery calcium
CAD: Coronary artery disease
CD-TLR: Clinically driven target lesion revascularization
CLTI: Chronic limb-threatening ischemia
CTA: Computed tomography angiography
DSA: Digital subtraction angiography
GLASS: Global limb anatomic staging system
IVUS: Intravascular ultrasound
MACE: Major adverse cardiovascular events
MALE: Major adverse limb events
(m)PACSS: (Modified) Peripheral arterial calcium scoring system
PA: Peroneal artery
PAD: Peripheral arterial disease
PARC: Peripheral academic research consortium
PCI: Percutaneous coronary intervention
TAC: Tibial artery calcification
TAP: Target arterial path
TPA: Tibial posterior artery
TPT: Tibioperoneal trunk
THRILLER: Dutch chronic lower limb-threatening ischemia registry
TL: Target lesion
TV: Target vessel
WIfI: Wound, Ischemia, foot Infection
