# Supplementary material for: Inter- and Intra-observer Agreement of the Peripheral Arterial Calcium Scoring System in Patients Undergoing (Infra)Popliteal Endovascular Interventions
Source: Cardiovasc Intervent Radiol. 2024 Aug 26;47(11):1441–9. doi: 10.1007/s00270-024-03839-1 (PMC11541408; doi:10.1007/s00270-024-03839-1)
Supplement: Supplementary file 1 — Supplementary file1 (DOCX 18 kb) [file 270_2024_3839_MOESM1_ESM.docx]

**Supplementary Table 1. Weighted kappa scores divided by a variety of peripheral arterial calcium scoring systems (PACSS) and treatment location.**

|  | **Inter-observer agreement^a^** | **Intra-observer agreement** |
| --- | --- | --- |
| **PACSS (0-4)** Popliteal  Infrapopliteal  Overall | 0.70 – 0.76  0.72 – 0.78  0.71 – 0.76 | 0.88  0.89  0.89 |
| **Binary PACSS (0-1)** Popliteal  Infrapopliteal  Overall | 0.68 – 0.83  0.72 – 0.78  0.68 – 0.74 | 0.80  0.90  0.86 |
| **TV calcification (0-2)** Popliteal  Infrapopliteal  Overall | 0.66 – 0.81  0.73 – 0.77  0.71 – 0.79 | 0.89  0.95  0.92 |
| **mPACSS (0-6)** Popliteal  Infrapopliteal  Overall | 0.71 – 0.75  0.73 – 0.78  0.72 – 0.77 | 0.88  0.91  0.90 |

Kappa results should be interpreted as follows: values 0.00 - 0.20 indicate slight agreement, 0.21-0.40 fair, 0.41-0.60 moderate, 0.61-0.80 substantial and 0.81-1.00 almost perfect agreement.

(m)PACSS = (modified) peripheral arterial calcium scoring system. TV = target vessel.

a. The results represent the range of weighted kappa values between two independent raters.
